# WHEN A LITTLE GOES A LONG WAY: EXPANDING HOME CARE SERVICES TO ADULTS WITH DISABILITIES

**DOI:** 10.1093/geroni/igz038.575

**Published:** 2019-11-08

**Authors:** Serena Hasworth, Jaclyn Winfree, Ozcan Tunalilar, Diana White

**Affiliations:** 1 Portland State University Institute on Aging, Portland, Oregon, United States; 2 Portland State University, Portland, Oregon, United States

## Abstract

Policy makers are increasingly interested in reducing public spending while maintaining quality of life. Since 1975, Oregon Project Independence (OPI) has supported community-based adults aged 60 and older to avoid or delay the need for residential long-term care services by increasing access to personal and home care services. The program also aims to prevent the need for Medicaid by optimizing personal resources and natural supports. In 2014, the OPI Expansion (OPI-E) pilot project began to serve adults aged 18-59 with disabilities in seven of Oregon’s seventeen Area Agencies on Aging (AAAs). This poster describes the evaluation of the expansion using three data sources: administrative data about consumer characteristics compiled by the AAAs and State of Oregon from 2015-2017 (N=3,824 traditional consumers, N= 581 OPI-E consumers), qualitative interviews conducted with AAA directors and OPI-E case managers (N=23), and a survey of current OPI-E consumers (N=126). Compared to traditional OPI consumers, OPI-E consumers were somewhat more likely to be men and people of color. Interviews with AAA staff highlighted the need for outreach, lack of service provider capacity, unique characteristics of younger consumers, and issues related to data management and rural access. Staff reported valuing the program, noting how “even low levels of service go a long way.” Qualitative and quantitative consumer responses showed consumers found OPI-E services invaluable. The majority stressed their appreciation for the program, with several describing it as “lifesaving.” These three sources informed recommendations for expanding the OPI-E program statewide.

